# Supplementary figures and images for: Genetic risk impacts stroke mortality and pathogenesis in patients with ischemic stroke: a cohort study of BioBank Japan
Source: Front Neurol. 2026 Feb 11;17:1664594. doi: 10.3389/fneur.2026.1664594 (PMC12932217; doi:10.3389/fneur.2026.1664594)

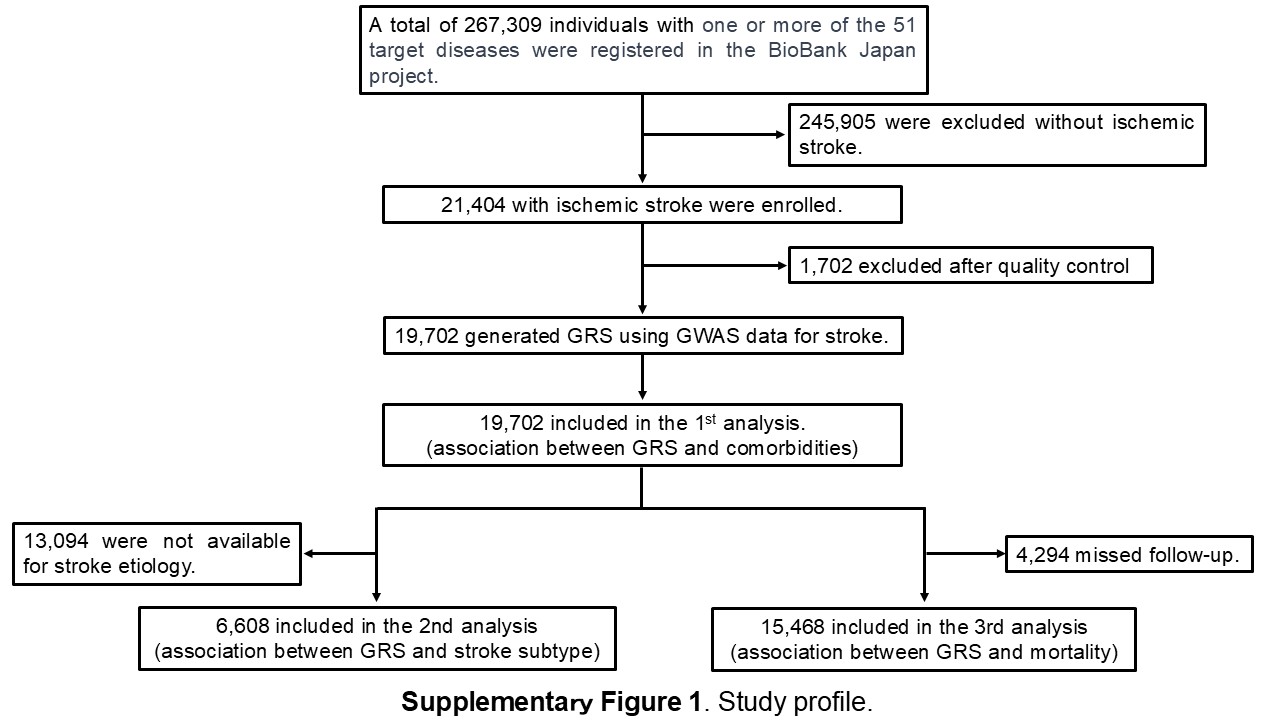

Supplement: Supplementary file 1 [file Image_1.jpeg]

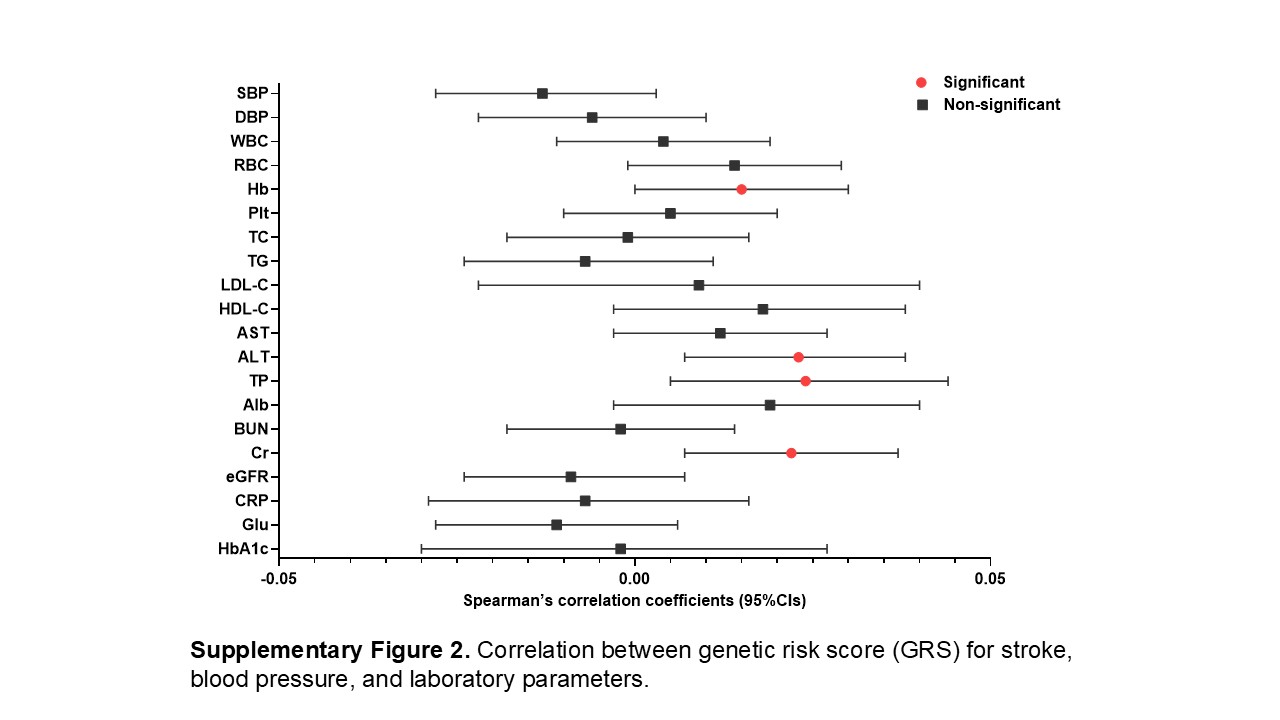

Supplement: Supplementary file 2 [file Image_2.jpeg]

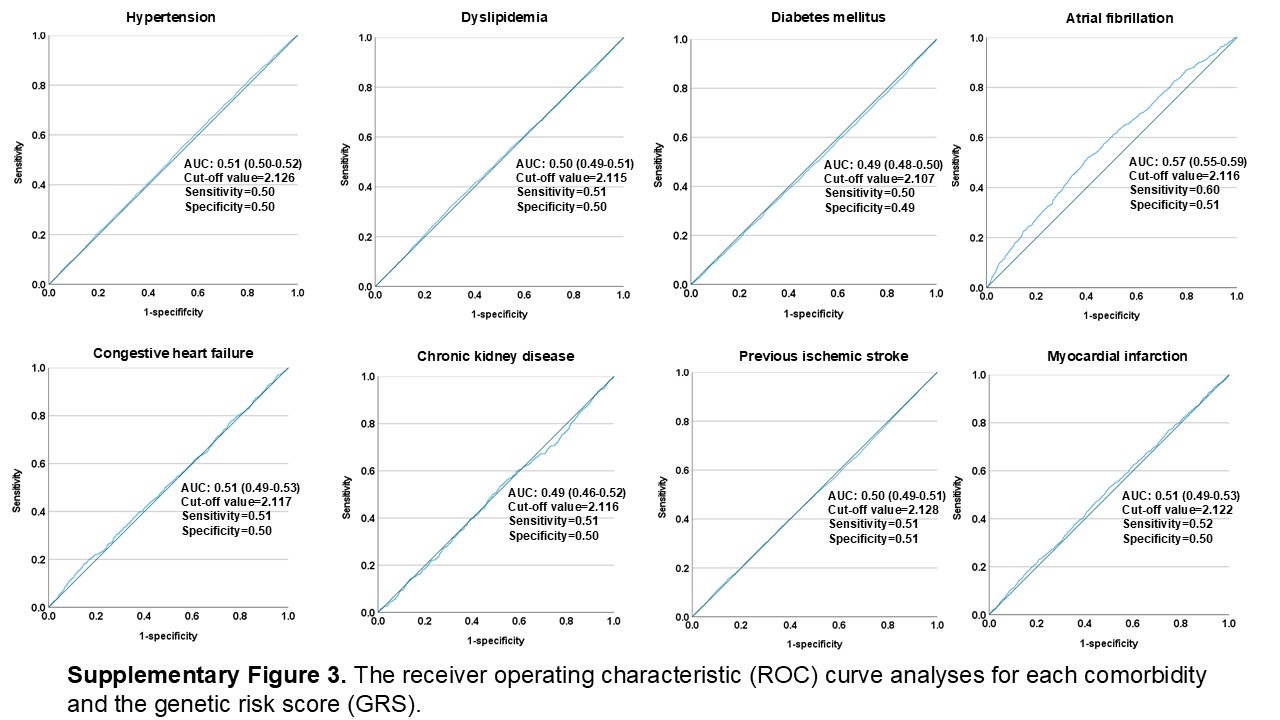

Supplement: Supplementary file 3 [file Image_3.jpeg]

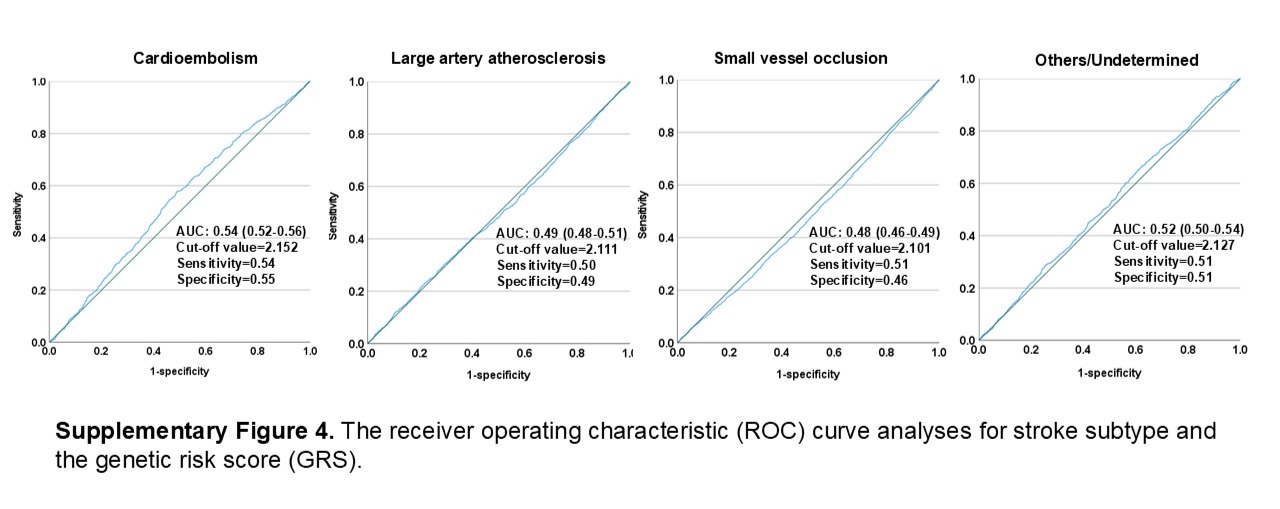

Supplement: Supplementary file 4 [file Image_4.jpeg]

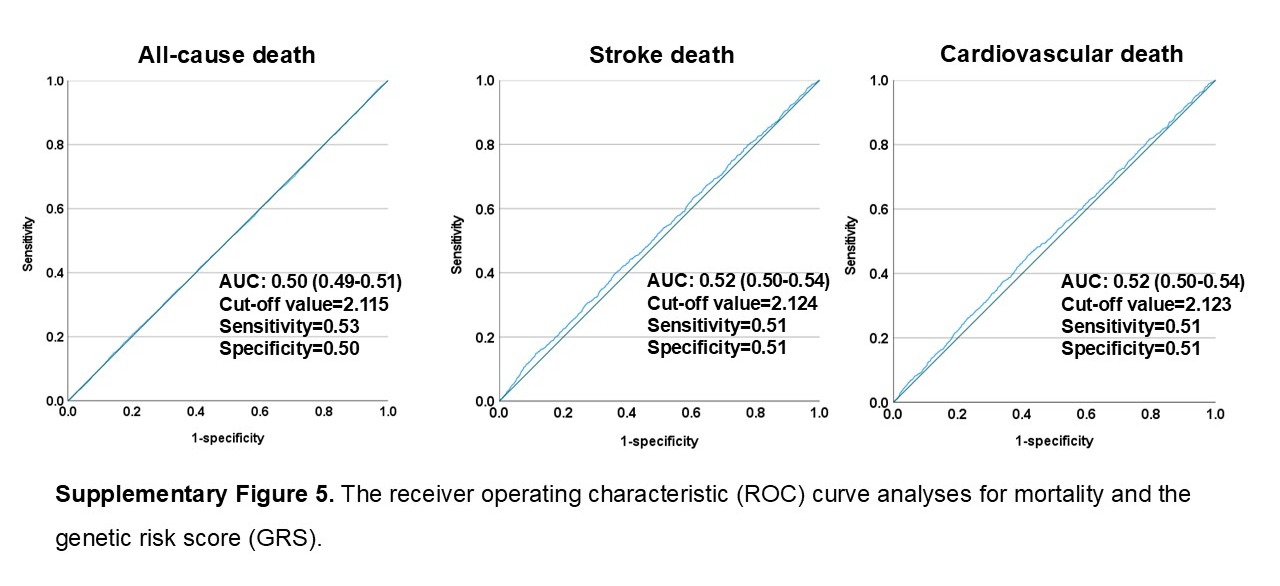

Supplement: Supplementary file 5 [file Image_5.jpeg]
