# Supplementary material for: Genetic risk impacts stroke mortality and pathogenesis in patients with ischemic stroke: a cohort study of BioBank Japan
Source: Front Neurol. 2026 Feb 11;17:1664594. doi: 10.3389/fneur.2026.1664594 (PMC12932217; doi:10.3389/fneur.2026.1664594)
Supplement: Supplementary file 6 [file Table_1.docx]

**Supplemental material**

**Supplementary Figure 1.** Study profile.

**Supplementary Figure 2.** Correlation between genetic risk score (GRS) for stroke, blood pressure, and laboratory parameters.

**Supplementary Figure 3.**The receiver operating characteristic (ROC) curve analyses for each comorbidity and the genetic risk score (GRS).

**Supplementary Figure 4.**The receiver operating characteristic (ROC) curve analyses for stroke subtype and the genetic risk score (GRS).

**Supplementary Figure 5.** The receiver operating characteristic (ROC) curve analyses for mortality and the genetic risk score (GRS).

**Supplementary Table 1.** Odds Ratios and 95% Confidential intervals for 32 stroke risk loci in MEGASTROKE and BioBank Japan (BBJ) cohorts.

**Supplementary Table 2.** Association between categories of genetic risk score (GRS) and baseline characteristics in the univariate analysis (n=19,702).

**Supplementary Table 3.** Association between categories of genetic risk score (GRS) and the prevalence of stroke subtype in the univariate analysis (n=6,608).

**Supplementary Table 4.** Association between MEGASTROKE genetic risk score (GRS) and comorbidities by multivariate logistic analysis (n=19,702).

**Supplemental Table 5.** Association between MEGASTROKE genetic risk score (GRS) and stroke etiology by multivariate logistic analysis (n=6,608).

**Supplemental Table 6.** Association between MEGASTROKE genetic risk score (GRS) and mortality by Cox proportional hazard analysis (n=15,468).

**Supplementary Figure legend.**

**Supplementary** **Figure 1.** Study profile.

**Supplementary Figure 2.** Correlation between genetic risk score (GRS) for stroke, blood pressure, and laboratory parameters. Data are presented as Spearman’s correlation coefficients and their 95% CIs. Significant correlations or associations are shown in red (*P< 0.05*).

SBP; systolic blood pressure, DBP; diastolic blood pressure, WBC; white blood cell, RBC; red blood cell, Hb; hemoglobin, Plt; platelet, AST; aspartate aminotransferase, ALT; alanine aminotransferase, BUN; blood urea nitrogen, Cr; creatinine, eGFR; **estimated glomerular filtration rate, TP; total protein, Alb;** albumin, TC; total cholesterol, LDL-C; low-density lipoprotein cholesterol, HDL-C; high-density lipoprotein cholesterol, TG; triglyceride, HbA1c; hemoglobin A1c.

**Supplementary Figure 3.**The receiver operating characteristic (ROC) curve analyses for each comorbidity and the genetic risk score (GRS).

**Supplementary Figure 4.**The receiver operating characteristic (ROC) curve analyses for stroke subtype and the genetic risk score (GRS).

**Supplementary Figure 5.** The receiver operating characteristic (ROC) curve analyses for mortality and the genetic risk score (GRS).

**Supplementary Table 1.** Odds Ratios and 95% Confidential intervals for 32 stroke risk loci in MEGASTROKE and BioBank Japan (BBJ) cohorts.

| **MEGASTROKE cohort** | | | | | | | | | | |  | **BBJ cohort** | | | |
| --- | --- | --- | --- | --- | --- | --- | --- | --- | --- | --- | --- | --- | --- | --- | --- |
| No | rsID | Chromosome | Gene | Risk allele/ reference allele | RAF (%) | Phenotype | Analysis | ORs | 95%CIs | *P* value |  | rsID | ORs | 95%CIs | *P* value |
| 1 | rs880315 | 1p36 | *CASZ1* | C/T | 40 | AS | TRANS | 1.05 | 1.04-1.07 | *3.62E-10* |  | rs880315 | 1.06 | 1.02-1.10 | *2.49E-03* |
| 2 | rs12037987 | 1p13 | *WNT2B* | C/T | 16 | AS | TRANS | 1.07 | 1.05-1.10 | *2.73E-08* |  | rs12037987 | 1.04 | 0.99-1.08 | *7.72E-02* |
| 3 | rs146390073 | 1q43 | *RGS7* | T/C | 2 | CE | EUR | 1.95 | 1.54-2.47 | *2.20E-08* |  | rs146390073 |  |  | *NA* |
| 4 | rs12476527 | 2p23 | *KCNK3* | G/T | 48 | AS | TRANS | 1.05 | 1.03-1.07 | *6.44E-08* |  | rs12476527 | 1.13 | 1.09-1.18 | *1.55E-09* |
| 5 | rs7610618 | 3q25 | *TM4SF4-TM4SF1* | T/C | 1 | LAA | EUR | 2.33 | 1.74-3.12 | *1.44E-08* |  | rs7610618 | 1.01 | 0.97-1.05 | *6.23E-01* |
| 6 | rs34311906 | 4q25 | *ANK2* | C/T | 41 | AIS | EUR | 1.07 | 1.04-1.09 | *1.07E-08* |  | rs34311906 | 1.01 | 0.97-1.05 | *6.23E-01* |
| 7 | rs17612742 | 4q31 | *EDNRA* | C/T | 21 | LAA | TRANS | 1.19 | 1.13-1.26 | *1.46E-11* |  | rs17612742 | 1.15 | 1.05-1.27 | *3.98E-03* |
| 8 | rs6825454 | 4q31 | *FGA* | C/T | 31 | AIS | TRANS | 1.06 | 1.04-1.08 | *7.43E-10* |  | rs6825454 | 1.04 | 1.00-1.08 | *4.57E-02* |
| 9 | rs11957829 | 5q23 | *LOC100505841* | A/G | 82 | AIS | TRANS | 1.07 | 1.05-1.10 | *7.51E-09* |  | rs11957829 | 1.17 | 1.04-1.34 | *1.52E-02* |
| 10 | rs6891174 | 5q35 | *NKX2-5* | A/G | 35 | CE | TRANS | 1.11 | 1.07-1.16 | *5.82E-09* |  | rs6891174 | 1.21 | 1.05-1.38 | *6.25E-03* |
| 11 | rs16896398 | 6p21 | *SLC22A7-ZNF318* | T/A | 34 | AS | TRANS | 1.05 | 1.03-1.07 | *1.30E-08* |  | rs16896398 | 1.05 | 1.01-1.09 | *1.21E-02* |
| 12 | rs42039 | 7q21 | *CDK6* | C/T | 77 | AIS | TRANS | 1.07 | 1.04-1.09 | *6.55E-09* |  | rs42039 | 0.95 | 0.83-1.09 | *4.61E-01* |
| 13 | rs7859727 | 9p21 | Chr9p21 | T/C | 53 | AS | TRANS | 1.05 | 1.03-1.07 | *4.22E-10* |  | rs7859727 | 1.07 | 1.03-1.11 | *3.92E-04* |
| 14 | rs10820405 | 9q31 | *LINC01492* | G/A | 82 | LAA | EUR | 1.20 | 1.12-1.28 | *4.51E-08* |  | rs10820405 | 1.05 | 0.95-1.15 | *3.17E-01* |
| 15 | rs2295786 | 10q24 | *SH3PXD2A* | A/T | 60 | AS | TRANS | 1.05 | 1.04-1.07 | *1.80E-10* |  | rs2295786 | 1.06 | 1.03-1.10 | *5.13E-04* |
| 16 | rs7304841 | 12p12 | *PDE3A* | A/C | 59 | AIS | TRANS | 1.05 | 1.03-1.07 | *4.93E-08* |  | rs7304841 | 1.07 | 1.03-1.11 | *3.92E-04* |
| 17 | rs35436 | 12q24 | *TBX3* | C/T | 62 | AS | TRANS | 1.05 | 1.03-1.06 | *2.87E-08* |  | rs35436 | 1.08 | 1.04-1.13 | *2.78E-04* |
| 18 | rs9526212 | 13q14 | *LRCH1* | G/A | 76 | AS | TRANS | 1.06 | 1.04-1.08 | *5.03E-10* |  | rs9526212 | 1.03 | 0.97-1.10 | *3.57E-01* |
| 19 | rs4932370 | 15q26 | *FURIN-FES* | A/G | 33 | AIS | TRANS | 1.05 | 1.03-1.07 | *2.88E-08* |  | rs4932370 | 1.07 | 1.00-1.14 | *4.30E-02* |
| 20 | rs11867415 | 17p13 | *PRPF8* | G/A | 18 | AIS | TRANS | 1.09 | 1.06-1.13 | *4.81E-08* |  | rs11867415 | 1.12 | 1.01-1.23 | *2.42E-02* |
| 21 | rs2229383 | 19p13 | *ILF3-SLC44A2* | T/G | 65 | AIS | TRANS | 1.05 | 1.03-1.07 | *4.72E-08* |  | rs2229383 | 1.03 | 0.99-1.07 | *1.36E-01* |
| 22 | rs8103309 | 19p13 | *SMARCA4-LDLR* | T/C | 65 | AS | TRANS | 1.05 | 1.03-1.07 | *3.40E-08* |  | rs8103309 | 1.06 | 1.00-1.12 | *4.39E-02* |
| 23 | rs12124533 | 1p13 | *TSPAN2* | T/C | 24 | LAA | TRANS | 1.17 | 1.11-1.23 | *1.22E-08* |  | rs12124533 |  |  | *NA* |
| 24 | rs1052053 | 1q22 | *PMF1-SEMA4A* | G/A | 40 | AS | TRANS | 1.06 | 1.05-1.08 | *2.70E-14* |  | rs1052053 | 1.04 | 1.01-1.08 | *2.18E-02* |
| 25 | rs13143308 | 4q25 | *PITX2* | T/G | 28 | CE | TRANS | 1.32 | 1.27-1.37 | *1.86E-47* |  | rs13143308 | 1.41 | 1.23-1.61 | *5.65E-07* |
| 26 | rs4959130 | 6p25 | *FOXF2* | A/G | 14 | AS | TRANS | 1.08 | 1.05-1.11 | *1.42E-09* |  | rs4959130 | 1.04 | 0.87-1.25 | *6.71E-01* |
| 27 | rs2107595 | 7p21 | *HDAC9-TWIST1* | A/G | 24 | LAA | TRANS | 1.21 | 1.15-1.26 | *3.65E-15* |  | rs2107595 | 1.09 | 0.99-1.19 | *6.64E-02* |
| 28 | rs635634 | 9q34 | *ABO* | T/C | 19 | AIS | EUR | 1.08 | 1.05-1.11 | *9.18E-09* |  | rs635634 |  |  | *NA* |
| 29 | rs2005108 | 11q22 | *MMP12* | T/C | 12 | AIS | TRANS | 1.08 | 1.05-1.11 | *3.33E-08* |  | rs2005108 | 1.14 | 1.02-1.28 | *2.37E-02* |
| 30 | rs3184504 | 12q24 | *SH2B3* | T/C | 45 | AIS | TRANS | 1.08 | 1.06-1.10 | *2.17E-14* |  | rs3184504 | 1.07 | 0.95-1.20 | *2.56E-01* |
| 31 | rs12932445 | 16q22 | *ZFHX3* | C/T | 21 | CE | TRANS | 1.20 | 1.15-1.25 | *6.86E-18* |  | rs12932445 | 1.21 | 1.07-1.36 | *1.83E-03* |
| 32 | rs12445022 | 16q24 | *ZCCHC14* | A/G | 31 | AS | TRANS | 1.06 | 1.04-1.08 | *1.05E-10* |  | rs12445022 | 1.08 | 1.02-1.15 | *1.19E-02* |

BBJ; BioBank Japan, RAF; risk-allele frequency, AS; any stroke, AIS, any ischemic stroke, CE; cardioembolism, LAA; large artery atherosclerosis, TRANS; transancestral meta-analysis, EUR; Europeans-only fixed-effects meta-analysis, ORs; odds ratios, CIs; confidence intervals.

**Supplementary Table 2.** Association between categories of genetic risk score (GRS) and baseline characteristics in the univariate analysis (n=19,702).

|  | Low GRS  (n=3,940) | Intermediate GRS  (n=11,823) | High GRS  (n=3,939) | *P* value |
| --- | --- | --- | --- | --- |
| Age, years-old; median (IQR) | 71 (64-77) | 71 (64-77) | 70 (63-77)**^a^** | *0.002* |
| Men, % | 61.2 | 63.4**^a^** | 64.2**^a^** | *0.013* |
| Hypertension, % | 35.5 | 37.2 | 38.0 | *0.050* |
| Dyslipidemia, % | 18.6 | 17.9 | 18.8 | *0.344* |
| Diabetes mellitus, % | 16.8 | 15.6 | 14.9 | *0.056* |
| Atrial fibrillation, % | 2.9 | 4.4**^a^** | 6.0**^a,b^** | *<0.001* |
| Congestive heart failure, % | 2.9 | 2.9 | 3.3 | *0.428* |
| Myocardial infarction, % | 4.6 | 4.5 | 5.0 | *0.434* |
| Chronic kidney failure, % | 2.4 | 2.0 | 1.9 | *0.294* |
| Previous ischaemic stroke, % | 44.6 | 43.3 | 43.8 | *0.342* |
| Smoking, % | 52.2 | 53.4 | 53.4 | *0.387* |
| Alcohol, % | 50.1 | 51.7 | 51.6 | *0.185* |

GRS; genetic risk score, Low (bottom 20^th^ percentile), Intermediate (20-80^th^ percentile), High (top 20^th^ percentile), IQR; interquartile

**a:** *P* value<.05 compared to Low GRS

**b:** *P* value<.05 compared to Intermediate GRS

**Supplementary Table 3.** Association between categories of genetic risk score (GRS) and the prevalence of stroke subtype in the univariate analysis (n=6608).

|  | Low GRS (n=1,321) | Intermediate GRS (n=3,966) | High GRS (n=1,321) | *P* value |
| --- | --- | --- | --- | --- |
| Stroke subtype |  |  |  |  |
| Cardioembolism (n=752), % | 8.9 | 11.8**^a^** | 12.7**^a^** | *0.005* |
| Large artery atherosclerosis (n=1,276), % | 20.4 | 18.7 | 20.0 | *0286* |
| Small vessel occlusion (n=3,657), % | 57.8**^b^** | 56.2**^b^** | 52.6 | *0.034* |
| Others/Undetermined (n=893), % | 12.9 | 13.3 | 14.7 | *0.458* |

GRS; genetic risk score, Low (bottom 20^th^ percent tertile), Intermediate (20-80^th^ percent tertile), High (top 20^th^ percent tertile).

**a:** *P value< 0.05* compared to Low GRS

**b:** *P value< 0.05* compared to High GRS

**Supplemental Table 4.** Association between MEGASTROKE genetic risk score (GRS) and comorbidities by multivariate logistic analysis (n=19,702).

|  | Model | Continuous MEGASTROKE GRS  (ORs: 95%CIs) | Low MEGASTREOKE GRS  (ORs: 95%CIs) | Intermediate MEGASTROKE GRS  (ORs: 95%CIs) | High MEGASTREOKE PRS  (ORs: 95%CIs) |
| --- | --- | --- | --- | --- | --- |
| Hypertension | 1 | 1.07 (0.99-1.15)  *P=0.058* | Ref | 1.05 (0.97-1.13)  *P=0.219* | 1.09 (0.99-1.20)  *P=0.058* |
|  | 2 | 1.07 (0.99-1.15)  *P=0.072* | Ref | 1.05 (0.98-1.13)  *P=0.196* | 1.10 (0.99-1.20)  *P=0.055* |
| Dyslipidemia | 1 | 1.02 (0.94-1.12)  *P=0.623* | Ref | 0.96 (0.87-1.05)  *P=0.348* | 1.02 (0.91-1.14)  *P=0.763* |
|  | 2 | 1.03 (0.94-1.13)  *P=0.530* | Ref | 1.00 (0.91-1.10)  *P=0.990* | 0.99 (0.88-1.12)  *P=0.867* |
| Diabetes mellitus | 1 | 0.92 (0.83-1.01)  *P=0.075* | Ref | 0.90 (0.82-0.99)  *P=0.041* | 0.86 (0.76-0.97)  *P=0.013* |
|  | 2 | 0.90 (0.82-0.99)  *P=0.040* | Ref | 0.92 (0.83-1.01)  *P=0.098* | 0.87 (0.77-0.99)  *P=0.036* |
| Atrial fibrillation | 1 | 1.41 (1.20-1.67)  *P<0.001* | Ref | 1.21 (1.00-1.46)  *P=0.051* | 1.42 (1.14-1.77)  *P=0.002* |
|  | 2 | 1.39 (1.18-1.65)  *P<0.001* | Ref | 1.22 (1.01-1.48)  *P=0.040* | 1.41 (1.13-1.77)  *P=0.002* |
| Congestive heart failure | 1 | 1.16 (0.95-1.42)  *P=0.140* | Ref | 1.02 (0.82-1.27)  *P=0.842* | 1.17 (0.91-1.52)  *P=0.219* |
|  | 2 | 1.06 (0.86-1.31)  *P=0.575* | Ref | 1.03 (0.82-1.29)  *P=0.817* | 1.12 (0.86-1.46)  *P=0.412* |
| Chronic kidney failure | 1 | 0.99 (0.78-1.26)  *P=0.934* | Ref | 0.84 (0.66-1.07)  *P=0.160* | 0.80 (0.59-1.09)  *P=0.155* |
|  | 2 | 0.98 (0.77-1.25)  *P=0.859* | Ref | 0.82 (0.64-1.05)  *P=0.109* | 0.91 (0.67-1.22)  *P=0.516* |
| Previous ischemic stroke | 1 | 0.97 (0.90-1.04)  *P=0.394* | Ref | 0.95 (0.89-1.03)  *P=0.206* | 0.95 (0.86-1.03)  *P=0.210* |
|  | 2 | 0.97 (0.90-1.04)  *P=0.339* | Ref | 0.96 (0.89-1.04)  *P=0.299* | 0.95 (0.87-1.04)  *P=0.281* |
| Myocardial infarction | 1 | 1.18 (1.00-1.39)  *P=0.045* | Ref | 1.04 (0.88-1.25)  *P=0.635* | 1.12 (0.91-1.39)  *P=0.281* |
|  | 2 | 1.17 (0.99-1.39)  *P=0.059* | Ref | 1.07 (0.89-1.28)  *P=0.462* | 1.14 (0.91-1.41)  *P=0.252* |

GRS; genetic risk score, Low GRS (bottom 20^th^ percentile), Intermediate GRS (20-80^th^ percentile), High GRS (top 20^th^ percentile), ORs; odds ratios, CIs; confidential intervals

Model 1; Sex and age-adjusted model, Model 2; Sex, age, comorbidities (hypertension, dyslipidemia, diabetes mellitus, atrial fibrillation, congestive heart failure, and chronic kidney failure), history of ischemic stroke and myocardial infarction, smoking, and alcohol consumption.

**Supplemental Table 5.** Association between MEGASTROKE genetic risk score (GRS) and stroke etiology by multivariate logistic analysis (n=6,608).

|  | Model | Continuous MEGASTROKE GRS  (ORs: 95%CIs) | Low MEGASTROKE GRS  (ORs: 95%CIs) | Intermediate MEGASTROKE GRS  (ORs: 95%CIs) | High MEGASTROKE GRS  (ORs: 95%CIs) |
| --- | --- | --- | --- | --- | --- |
| Cardioembolism | 1 | 1.12 (0.93-1.34)  *P=0.248* | Ref | 1.28 (1.04-1.57)  *P=0.022* | 1.19 (0.93-1.53)  *P=0.166* |
|  | 2 | 1.10 (0.90-1.33)  *P=0.353* | Ref | 1.25 (1.00-1.55)  *P=0.047* | 1.19 (0.92-1.54)  *P=0.194* |
| Large artery atherosclerosis | 1 | 0.96 (0.83-1.18)  *P=0.603* | Ref | 0.96 (0.82-1.13)  *P=0.631* | 1.02 (0.84-1.23)  *P=0.863* |
|  | 2 | 0.97 (0.83-1.13)  *P=0.677* | Ref | 0.97 (0.83-1.14)  *P=0.708* | 1.02 (0.85-1.24)  *P=0.809* |
| Small vessel occlusion | 1 | 0.84 (0.75-0.95)  *P=0.005* | Ref | 0.90 (0.79-1.02)  *P=0.096* | 0.81 (0.69-0.95)  *P=0.008* |
|  | 2 | 0.85 (0.75-0.96)  *P=0.007* | Ref | 0.90 (0.79-1.03)  *P=0.117* | 0.81 (0.69-0.95)  *P=0.007* |
| Others/Undetermined | 1 | 1.36 (1.15-1.61)  *P<0.001* | Ref | 1.09 (0.90-1.31)  *P=0.398* | 1.31 (1.05-1.63)  *P=0.018* |
|  | 2 | 1.36 (1.15-1.61)  *P<0.001* | Ref | 1.09 (0.90-1.31)  *P=0.403* | 1.31 (1.05-1.63)  *P=0.019* |

GRS; genetic risk score, Low GRS (bottom 20th percentile), Intermediate GRS (20-80th percentile), High GRS (top 20th percentile), ORs; odds ratios, CIs; confidential intervals.

Model 1; Sex and age-adjusted model, Model 2; Sex, age, comorbidities (hypertension, dyslipidemia, diabetes mellitus, atrial fibrillation, congestive heart failure, and chronic kidney failure), history of ischemic stroke and myocardial infarction, smoking, and alcohol consumption.

**Supplemental Table 6.** Association between MEGASTROKE genetic risk score (GRS) and mortality by Cox proportional hazard analysis (n=15,468).

|  | No of events (%) | Model | Continuous　MEGASTROKE　GRS  (HRs: 95%CIs) | Low MEGASTREOKE GRS  (HRs: 95%CIs) | Intermediate MEGASTROKE　GRS  (HRs: 95%CIs) | High MEGASTROKE GRS  (HRs: 95%CIs) |
| --- | --- | --- | --- | --- | --- | --- |
| All-cause mortality | 6,253 (31.7%) | 1 | 1.07 (1.01-1.14)  *P=0.026* | Ref | 1.02 (0.95-1.09)  *P=0.596* | 1.03 (0.95-1.12)  *P=0.576* |
|  |  | 2 | 1.06 (0.99.-1.13)  *P=0.074* | Ref | 1.01 (0.95-1.08)  *P=0.707* | 1.02 (0.95-1.11)  *P=0.063* |
| Stroke mortality | 949 (4.8%) | 1 | 1.02 (0.94-1.12)  *P=0.623* | Ref | 0.97 (0.82-1.14)  *P=0.675* | 0.96 (0.79-1.17)  *P=0.695* |
|  |  | 2 | 1.06 (0.90-1.24)  *P=0.496* | Ref | 0.96 (0.82-1.13)  *P=0.643* | 0.96 (0.78-1.17)  *P=0.654* |
| CV mortality | 1,167 (5.9%) | 1 | 1.32 (1.15-1.52)  *P<0.001* | Ref | 1.14 (0.98-1.33)  *P=0.094* | 1.29 (1.07-1.55)  *P=0.006* |
|  |  | 2 | 1.28 (1.11-1.47)  *P<0.001* | Ref | 1.13 (0.96-1.31)  *P=0.134* | 1.26 (1.05-1.51)  *P=0.014* |

GRS; genetic risk score, Low GRS (bottom 20^th^ percentile), Intermediate GRS (20-80^th^ percentile), High GRS (top 20^th^ percentile), HRs; hazard ratios, CIs; confidential intervals, CV; cardiovascular.

Model 1; Sex and age-adjusted model, Model 2; Sex, age, comorbidities (hypertension, dyslipidemia, diabetes mellitus, atrial fibrillation, congestive heart failure, and chronic kidney failure), history of ischemic stroke and myocardial infarction, smoking, and alcohol consumption.
